# Integrative Analysis of Transcriptome-Wide Association Study and mRNA Expression Profiles Identifies Candidate Genes Associated With Idiopathic Pulmonary Fibrosis

**DOI:** 10.3389/fgene.2020.604324

**Published:** 2020-12-10

**Authors:** Weiming Gong, Ping Guo, Lu Liu, Qingbo Guan, Zhongshang Yuan

**Affiliations:** ^1^Department of Biostatistics, School of Public Health, Cheeloo College of Medicine, Shandong University, Jinan, China; ^2^Department of Endocrinology, Shandong Provincial Hospital Affiliated to Shandong First Medical University, Jinan, China; ^3^Shandong Clinical Medical Center of Endocrinology and Metabolism, Jinan, China; ^4^Shandong Institute of Endocrine and Metabolic Diseases, Jinan, China

**Keywords:** idiopathic pulmonary fibrosis, transcriptome-wide association study, gene expression profiling, pathway enrichment, protein–protein interaction network

## Abstract

Idiopathic pulmonary fibrosis (IPF) is a type of scarring lung disease characterized by a chronic, progressive, and irreversible decline in lung function. The genetic basis of IPF remains elusive. A transcriptome-wide association study (TWAS) of IPF was performed by FUSION using gene expression weights of three tissues combined with a large-scale genome-wide association study (GWAS) dataset, totally involving 2,668 IPF cases and 8,591 controls. Significant genes identified by TWAS were then subjected to gene ontology (GO) and pathway enrichment analysis. The overlapped GO terms and pathways between enrichment analysis of TWAS significant genes and differentially expressed genes (DEGs) from the genome-wide mRNA expression profiling of IPF were also identified. For TWAS significant genes, protein–protein interaction (PPI) network and clustering modules analyses were further conducted using STRING and Cytoscape. Overall, TWAS identified a group of candidate genes for IPF under the Bonferroni corrected *P* value threshold (0.05/14929 = 3.35 × 10^–6^), such as *DSP* (*P*_*TWAS*_ = 1.35 × 10^–29^ for lung tissue), *MUC5B* (*P*_*TWAS*_ = 1.09 × 10^–28^ for lung tissue), and *TOLLIP* (*P*_*TWAS*_ = 1.41 × 10^–15^ for whole blood). Pathway enrichment analysis identified multiple candidate pathways, such as herpes simplex infection (*P* value = 7.93 × 10^–5^) and antigen processing and presentation (*P* value = 6.55 × 10^–5^). 38 common GO terms and 8 KEGG pathways shared by enrichment analysis of TWAS significant genes and DEGs were identified. In the PPI network, 14 genes (*DYNLL1*, *DYNC1LI1*, *DYNLL2*, *HLA-DRB5*, *HLA-DPB1*, *HLA-DQB2*, *HLA-DQA2*, *HLA-DQB1*, *HLA-DRB1*, *POLR2L*, *CENPP*, *CENPK*, *NUP133*, and *NUP107*) were simultaneously detected by hub gene and module analysis. In conclusion, through integrative analysis of TWAS and mRNA expression profiles, we identified multiple novel candidate genes, GO terms and pathways for IPF, which contributes to the understanding of the genetic mechanism of IPF.

## Introduction

Idiopathic pulmonary fibrosis (IPF) is a chronic interstitial lung disease characterized by the formation of scar tissue and a progressive, and irreversible decline in lung function (Raghu et al., 2011; Lederer and Martinez, 2018), with a median survival time from diagnosis of 2–4 years (Ley et al., 2011). The incidence of IPF is increasing worldwide and has been estimated to be 3–9 cases per 100,000 people per year in Europe and North America, and fewer than four cases per 100,000 people per year in East Asia and South America (Hutchinson et al., 2015). IPF has been confirmed to be related to varieties of environmental and genetic factors. Potential risk factors for IPF include aging, male sex, smoking, certain occupational exposures (Baumgartner et al., 2000), gastroesophageal reflux (Tobin et al., 1998; Bedard Methot et al., 2019), herpesvirus infection (Tang et al., 2003), air pollution (Sack et al., 2017), and obstructive sleep apnea (Kim et al., 2017). Genome-wide association studies (GWAS) on IPF (Mushiroda et al., 2008; Fingerlin et al., 2013, 2016; Noth et al., 2013; Allen et al., 2017, 2020) have identified common genetic variants related to IPF, highlighting the significance of several IPF susceptibility factors, such as telomere maintenance, host defense, cell-cell adhesion. Rare genetic variants regarding surfactant dysfunction and telomere biology have also been identified in studies of familial pulmonary fibrosis (Nogee et al., 2001; Armanios et al., 2007; Cogan et al., 2015; Stuart et al., 2015).

Genome-wide association study have significantly succeeded in identifying IPF-related susceptibility genetic loci. However, a great number of genetic variations identified reside in non-coding regions, which are generally difficult to characterize biologically. Indeed, one common sense for GWAS is that most disease-associated genetic variants are located in non-coding regions, resulting in the hypothesis that the underlying biological mechanism of disease may be closely related to gene expression regulation. Furthermore, several expression quantitative trait loci (eQTLs) studies have illustrated that the information on expression regulation may play a pivotal role in disease development (Albert and Kruglyak, 2015). Transcriptome-wide association study (TWAS) is widely utilized in integrating GWAS with eQTL studies for investigating the causal genes associated with complex traits or diseases (Gamazon et al., 2015; Gusev et al., 2016; Yuan et al., 2020). Therefore, TWAS analysis may help us to identify novel genes associated with IPF. On the other hand, the genome-wide mRNA expression profiling of IPF provides the opportunity to identify differentially expressed genes (DEGs). Furthermore, omics integrative analysis can combine different types of omics data and provides more comprehensive insights than that offered by any single type of omics data (Liu et al., 2013). These integrative analyses are implemented and expected to rebuild meaningful biological networks by integrating information from different types of data, thus have the potential to provide a more novel and reliable understanding with respect to the underlying biological mechanisms. Statistically, complementary information can be better captured and exploited by such data integration analyses (Yu and Zeng, 2018). Indeed, in high-throughput genomic studies, one common sense is that the analysis from single dataset often lack of reproducibility and integrative analysis can efficiently investigate and make full use of multiple datasets in a cost-efficient manner to enhance reproducibility (Yu and Zeng, 2018). This motivated us to perform a comprehensive integrative analysis of TWAS and mRNA expression profile of IPF, which may provide the better understanding of the molecular mechanisms of IPF.

**FIGURE 1:**
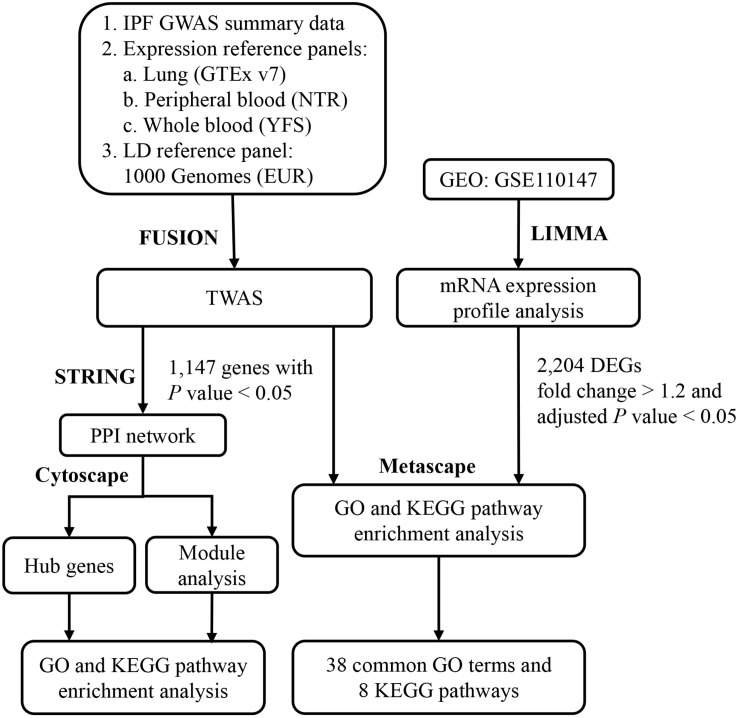
The flowchart illustrates the procedure of integrative analysis of TWAS and mRNA expression profile of IPF. Software for the integrative analysis were shown in bold. IPF, idiopathic pulmonary fibrosis; GWAS, genome-wide association study; GTEx, Genotype-Tissue Expression Project; NTR, Netherlands Twin Registry study; YFS, Young Finns Study; EUR, European; LD, linkage disequilibrium; TWAS, transcriptome-wide association study; GEO, Gene Expression Omnibus database; DEGs, differentially expressed genes; STRING, Search Tool for the Retrieval of Interacting Genes; PPI, protein–protein interaction; GO, gene ontology; KEGG, Kyoto Encyclopedia of Genes and Genomes.

In the present study, we leveraged expression imputation from a large-scale IPF GWAS dataset to perform a TWAS analysis in peripheral blood, whole blood and lung tissue. The TWAS significant genes and DEGs identified by mRNA expression profiling of IPF were then subjected to gene ontology (GO) and Kyoto Encyclopedia of Genes and Genomes (KEGG) pathway enrichment analysis. Through this analysis, the common GO terms and KEGG pathways were identified. Furthermore, for significant genes identified by TWAS for IPF, STRING and Cytoscape software were applied to implement protein–protein interaction (PPI) network and clustering modules analyses. Our results may provide novel insights into the understanding of the molecular mechanisms underlying the development of IPF. The detailed procedure of integrative analysis was displayed in Figure 1.

## Materials and Methods

### GWAS of IPF

The current and largest-scale GWAS summary data of IPF were used (Allen et al., 2020). Briefly, it included 2,668 IPF cases and 8,591 controls of European ancestry from a meta-analysis of three case-control studies and restricted to unrelated individuals of European ancestry. Genotype data were imputed using the Haplotype Reference Consortium r1.1 panel. Stringent quality control was performed for the genotyped data. In each separate study, a genome-wide analysis of IPF susceptibility was conducted using SNPTEST v2.5.2, adjusting for the first 10 principal components to account for fine-scale population structure. Only biallelic autosomal variants that had a minor allele count ≥10, were in Hardy-Weinberg Equilibrium (*P* > 1 × 10^–6^), and well-imputed (imputation quality *R*^2^ > 0.5) in at least two studies were included. Detailed description related to study participants, genotyping, imputation, association analysis, and quality control can be found in the previous IPF GWAS study (Allen et al., 2020).

### TWAS of IPF

FUSION software was applied here for tissue-related TWAS analysis (Gusev et al., 2016). Briefly, FUSION leveraged a set of reference individuals to measure both gene expression and SNPs, and then to impute the *cis* genetic component of expression into a much larger set of individuals using their SNP genotype data. The imputed expression data can be viewed as a linear model of genotypes with weights based on the correlation between SNPs and gene expression in the reference data while accounting for linkage disequilibrium among SNPs. FUSION uses pre-computed gene expression weights together with disease GWAS summary statistics to evaluate the association between the expression levels of genes and target diseases (Gusev et al., 2016). The genetic values of expression were computed as one probe set at a time using SNP genotyping data located 500 kb on both sides of the gene boundary. The pre-computed expression reference weights of different tissues were downloaded from the FUSION websites^1^. For IPF TWAS, we used three expression reference panels, including lung, peripheral blood and whole blood, and a TWAS *P* value was obtained for each gene. Gene expression weights of lung were driven from the Genotype-Tissue Expression Project (GTEx v7; *n* = 383) (GTEx Consortium et al., 2017). Gene expression weights of peripheral blood and whole blood reference panels were driven from the Netherlands Twin Registry study (NTR) (*n* = 1,247) (Boomsma et al., 2006; Wright et al., 2014) and Young Finns Study (YFS) (*n* = 1,264) (Raitakari et al., 2008), respectively.

### Gene Expression Profile Associated With IPF

The IPF gene expression profile data of lung tissue were obtained from the Gene Expression Omnibus database (access number: GSE110147) (Cecchini et al., 2018). Briefly, fresh frozen lung samples were obtained from the organs of 22 patients with IPF; normal lung tissue (*n* = 11) was obtained from the tissue flanking lung cancer resections. RNA was extracted and hybridized on Affymetrix microarrays. Individual-level gene expression data were included in the mRNA expression profile analysis implemented by LIMMA package (Ritchie et al., 2015). The DEGs between IPF patients and controls were identified at fold change >1.2 and adjusted *P* value < 0.05. Detailed description of sample characteristics, experimental design, statistical analysis, and quality control can be found in the previous study (Cecchini et al., 2018).

### Gene Set Enrichment Analysis

The IPF-related genes identified by TWAS and mRNA expression profiling were, respectively, subjected to GO and KEGG pathway enrichment analysis implemented by Metascape (Zhou et al., 2019)^2^. Note that in the enrichment analysis for IPF-related genes identified by TWAS, we included all the genes with a TWAS *P* value less than 0.05, rather than those under the Bonferroni corrected *P* value threshold (0.05/14929 = 3.35 × 10^–6^), to increase the ability to identify more biological processes relevant to IPF and to make the results more stable by including more input genes (Reimand et al., 2019). A *P* value was calculated by Metascape for each GO term and pathway. Terms with a *P* value < 0.01, a minimum count of 3, and an enrichment factor >1.5 were collected and grouped into clusters based on their membership similarities. Kappa scores were used as the similarity metric when performing hierarchical clustering on the enriched terms, and sub-trees with a similarity of >0.3 were considered a cluster. The most statistically significant term within a cluster was chosen to represent the cluster. Finally, the Metascape analysis of TWAS was compared with that of mRNA expression profiles of lung tissue to identify the common GO terms and pathways shared by enrichment analysis for IPF-related genes from TWAS and for DEGs from mRNA expression profiling of IPF. Note that the common GO terms were obtained by overlapping the original GO enrichment results before grouping into clusters.

### Protein–Protein Interaction Network, Hub Genes, and Module Analysis

The PPI network of TWAS significant genes was constructed by the online Search Tool for the Retrieval of Interacting Genes (Szklarczyk et al., 2019) (STRING; 2017 release) database to evaluate the interactive relationships among the genes. Interactions with a combined score >0.9 were defined as statistically significant. Cytoscape software (Shannon et al., 2003) (version 3.5.1) was applied to visualize the integrated regulatory networks. The cytoHubba plugin and Molecular Complex Detection (MCODE) plugin in Cytoscape were used to identify hub genes and screen modules of the PPI network. All parameters of the plugin were set at their default values. Again, GO and KEGG enrichment of hub genes and genes in modules were also analyzed by Metascape.

## Results

### TWAS Analysis Results

Totally, 14,929 genes were analyzed by TWAS in this study. Overall, TWAS identified 29 genes under the Bonferroni corrected *P* value threshold (0.05/14929 = 3.35 × 10^–6^) and 1,147 genes with *P* value < 0.05 (Supplementary Table S1), such as *DSP* (*P*_*TWAS*_ = 1.35 × 10^–29^ for lung tissue), *MUC5B* (*P*_*TWAS*_ = 1.09 × 10^–28^ for lung tissue), *TOLLIP* (*P*_*TWAS*_ = 1.41 × 10^–15^ for whole blood), *MAPT* (*P*_*TWAS*_ = 9.60 × 10^–15^ for lung tissue), and *DEPTOR* (*P*_*TWAS*_ = 8.58 × 10^–9^ for lung tissue). The top 30 genes identified by TWAS are summarized in Table 1.

**TABLE 1:** Top 30 genes identified by TWAS for IPF.

Gene	Chromosome	*P*_*TWAS*_	Tissue
*DSP*	6	1.35 × 10 ^–29^	Lung
*MUC5B*	11	1.09 × 10^–28^	Lung
*TOLLIP*	11	1.41 × 10^–15^	Whole blood
*DND1P1*	17	8.12 × 10^–15^	Lung
*CRHR1-IT1*	17	9.20 × 10^–15^	Lung
*MAPT*	17	9.60 × 10^–15^	Lung
*RP11-259G18.2*	17	1.04 × 10^–14^	Lung
*RP11-707O23.5*	17	1.19 × 10^–14^	Lung
*RP11-259G18.3*	17	1.22 × 10^–14^	Lung
*LRRC37A4P*	17	1.59 × 10^–14^	Lung
*KANSL1-AS1*	17	2.75 × 10^–14^	Lung
*RP11-259G18.1*	17	3.81 × 10^–14^	Lung
*KIAA1267*	17	2.19 × 10^–13^	Peripheral blood
*LRRC37A2*	17	2.91 × 10^–13^	Lung
*WNT3*	17	1.21 × 10^–12^	Lung
*DND1*	17	2.17 × 10^–12^	Peripheral blood
*PLEKHM1*	17	5.00 × 10^–11^	Peripheral blood
*FAM215B*	17	7.66 × 10^–11^	Lung
*PLEKHM1*	17	3.08 × 10^–10^	Lung
*RP11-158M2.5*	15	1.41 × 10^–9^	Lung
*FAM13A*	4	2.12 × 10^–9^	Lung
*LRRC37A*	17	6.40 × 10^–9^	Lung
*DEPTOR*	8	8.58 × 10^–9^	Lung
*RP11-760H22.2*	8	1.06 × 10^–8^	Lung
*BAHD1*	15	1.16 × 10^–8^	Lung
*BRSK2*	11	1.49 × 10^–8^	Lung
*RP11-798G7.5*	17	2.97 × 10^–8^	Lung
*GCHFR*	15	1.94 × 10^–7^	Whole blood
*RP11-64K12.8*	15	2.37 × 10^–7^	Lung
*ZNF514*	2	4.41 × 10^–6^	Whole blood

### Gene Set Enrichment Analysis

**FIGURE 2:**
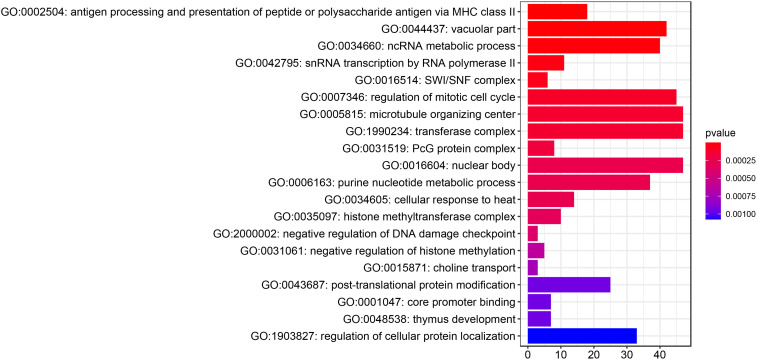
The top 20 gene ontology terms identified by enrichment analysis for IPF-related genes from TWAS.

**FIGURE 3:**
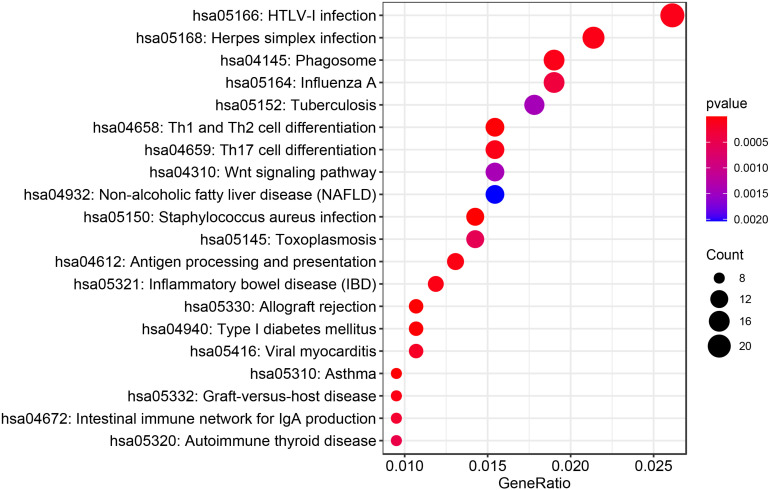
The top 20 KEGG pathways identified by enrichment analysis for IPF-related genes from TWAS.

A total of 1,147 genes with a TWAS *P* value < 0.05 were included in the GO enrichment analysis. Metascape detected 76 GO terms under *P* value < 0.01 (Figure 2 and Supplementary Table S2), such as antigen processing and presentation of peptide or polysaccharide antigen via major histocompatibility complex (MHC) class II (*P*_*TWAS*_ = 1.43 × 10^–8^), vacuolar part (*P*_*TWAS*_ = 5.98 × 10^–6^), ncRNA metabolic process (*P*_*TWAS*_ = 1.61 × 10^–5^), snRNA transcription by RNA polymerase II (*P*_*TWAS*_ = 3.03 × 10^–5^), and SWI/SNF complex (*P*_*TWAS*_ = 5.81 × 10^–5^). For KEGG pathway enrichment analysis of the genes identified by TWAS, Metascape detected 30 candidate pathways for IPF under *P* value < 0.01 (Figure 3 and Supplementary Table S3), such as *Staphylococcus aureus* infection (*P*_*TWAS*_ = 3.69 × 10^–7^), allograft rejection (*P*_*TWAS*_ = 4.45 × 10^–6^), asthma (*P*_*TWAS*_ = 7.65 × 10^–6^), type I diabetes mellitus (*P*_*TWAS*_ = 1.32 × 10^–5^), inflammatory bowel disease (IBD) (*P*_*TWAS*_ = 7.37 × 10^–5^), and herpes simplex infection (*P*_*TWAS*_ = 7.93 × 10^–5^).

A total of 2,204 DEGs were identified by mRNA expression profiling analysis of IPF (Supplementary Table S4), and were then conducted GO and KEGG pathway enrichment analysis (Supplementary Tables S5, S6). 38 common GO terms were identified by enrichment analysis of IPF-related genes identified by TWAS and DEGs (Supplementary Table S7), including antigen processing and presentation of peptide or polysaccharide antigen via MHC class II (GO:0002504, *P*_*TWAS*_ = 1.43 × 10^–8^, *P*_*mRNA*_ = 8.87 × 10^–3^), lytic vacuole (GO:0000323, *P*_*TWAS*_ = 6.25 × 10^–6^, *P*_*mRNA*_ = 5.91 × 10^–3^), ncRNA metabolic process (GO:0034660, *P*_*TWAS*_ = 1.61 × 10^–5^, *P*_*mRNA*_ = 4.43 × 10^–7^), microtubule organizing center (GO:0005815, *P*_*TWAS*_ = 1.24 × 10^–4^, *P*_*mRNA*_ = 2.46 × 10^–23^), and autophagy (GO:0002504, *P*_*TWAS*_ = 1.10 × 10^–3^, *P*_*mRNA*_ = 2.11 × 10^–4^). Table 2 summarizes the top 20 common GO terms detected by enrichment analysis of IPF-related genes identified by TWAS and DEGs. We also detected 8 common KEGG pathways (Table 3), such as staphylococcus aureus infection (*P*_*TWAS*_ = 3.69 × 10^–7^, *P*_*mRNA*_ = 9.17 × 10^–3^), herpes simplex infection (*P*_*TWAS*_ = 7.93 × 10^–5^, *P*_*mRNA*_ = 8.76 × 10^–4^), HTLV-I infection (*P*_*TWAS*_ = 8.84 × 10^–5^, *P*_*mRNA*_ = 5.92 × 10^–4^), phagosome (*P*_*TWAS*_ = 8.99 × 10^–5^, *P*_*mRNA*_ = 1.93 × 10^–4^), and systemic lupus erythematosus (*P*_*TWAS*_ = 2.25 × 10^–3^, *P*_*mRNA*_ = 3.12 × 10^–3^).

**TABLE 2:** Top 20 overlapped gene ontology terms identified by enrichment analysis for IPF-related genes from TWAS and for differentially expressed genes from mRNA expression profiling of IPF.

ID	Category	Description	*P*_*TWAS*_	*P*_*mRNA*_
GO:0002504	BP	Antigen processing and presentation of peptide or polysaccharide antigen via MHC class II	1.43 × 10^–8^	8.87 × 10^–3^
GO:0000323	CC	Lytic vacuole	6.25 × 10^–6^	5.91 × 10^–3^
GO:0034660	BP	ncRNA metabolic process	1.61 × 10^–5^	4.43 × 10^–7^
GO:0042795	BP	snRNA transcription by RNA polymerase II	3.03 × 10^–5^	8.88 × 10^–3^
GO:0006338	BP	Chromatin remodeling	2.47 × 10^–4^	1.37 × 10^–5^
GO:0007346	BP	Regulation of mitotic cell cycle	9.76 × 10^–5^	1.43 × 10^–12^
GO:0005815	CC	Microtubule organizing center	1.24 × 10^–4^	2.46 × 10^–23^
GO:1990234	CC	Transferase complex	1.32 × 10^–4^	9.74 × 10^–6^
GO:0016604	CC	Nuclear body	2.50 × 10^–4^	2.37 × 10^–16^
GO:0006163	BP	Purine nucleotide metabolic process	2.56 × 10^–4^	2.17 × 10^–6^
GO:0034605	BP	Cellular response to heat	2.68 × 10^–4^	4.12 × 10^–3^
GO:0044417	BP	Translocation of molecules into host	2.95 × 10^–3^	5.04 × 10^–4^
GO:0043687	BP	Post-translational protein modification	9.48 × 10^–4^	8.44 × 10^–6^
GO:1903827	BP	Regulation of cellular protein localization	1.08 × 10^–3^	2.58 × 10^–6^
GO:0006914	BP	Autophagy	1.10 × 10^–3^	2.11 × 10^–4^
GO:0098687	CC	Chromosomal region	1.31 × 10^–3^	7.26 × 10^–15^
GO:0072594	BP	Establishment of protein localization to organelle	1.55 × 10^–3^	6.00 × 10^–5^
GO:0008134	MF	Transcription factor binding	1.55 × 10^–3^	4.44 × 10^–4^
GO:0032984	BP	Protein-containing complex disassembly	3.84 × 10^–3^	7.07 × 10^–3^
GO:0000139	CC	Golgi membrane	1.87 × 10^–3^	7.40 × 10^–6^

*Biological Processes (BP); Cellular Components (CC); Molecular Functions (MF).*

**TABLE 3:** Overlapped KEGG pathways identified by enrichment analysis for IPF-related genes from TWAS and for differentially expressed genes from mRNA expression profiling of IPF.

ID	Description	*P*_*TWAS*_	*P*_*mRNA*_
hsa05150	Staphylococcus aureus infection	3.69 × 10^–7^	9.17 × 10^–3^
hsa05168	Herpes simplex infection	7.93 × 10^–5^	8.76 × 10^–4^
hsa05166	HTLV-I infection	8.84 × 10^–5^	5.92 × 10^–4^
hsa04145	Phagosome	8.99 × 10^–5^	1.93 × 10^–4^
hsa05164	Influenza A	3.46 × 10^–4^	1.32 × 10^–3^
hsa04932	Non-alcoholic fatty liver disease (NAFLD)	2.04 × 10^–3^	2.64 × 10^–4^
hsa05322	Systemic lupus erythematosus	2.25 × 10^–3^	3.12 × 10^–3^
hsa04620	Toll-like receptor signaling pathway	9.97 × 10^–3^	9.76 × 10^–3^

### PPI Network, Hub Gene and Module Analysis

**FIGURE 4:**
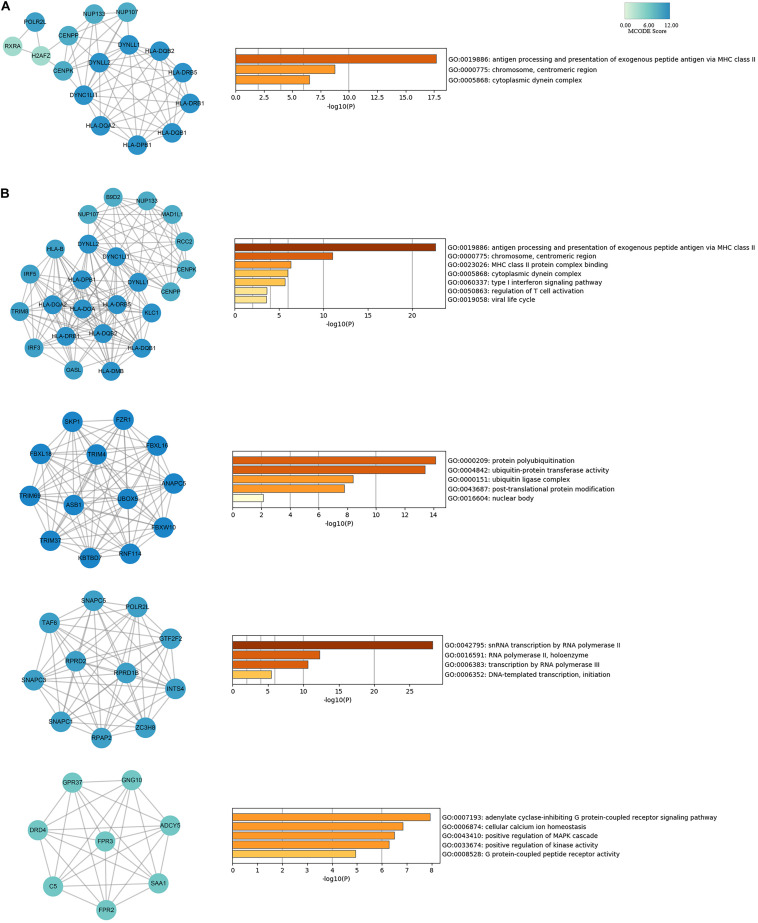
PPI network for hub genes and modules analyses of IPF-related genes identified by TWAS. **(A)** The network of 16 hub genes with a higher degree of connectivity and enrichment analysis of these genes. **(B)** Genes of top four modules were subjected to GO and KEGG enrichment analysis by Metascape.

To evaluate the association of IPF-related genes identified by TWAS, a PPI network was constructed by STRING and visualized by Cytoscape, containing 329 nodes and 893 edges (Supplementary Figure S1). The top 20 hub genes were identified by cytoHubba plugin that uses 12 different algorithms (Supplementary Table S8). Then, from the genes that can be detected by more than five algorithms, 16 hub genes with the highest degree of connectivity were selected to build the hub gene PPI network (Figure 4A). The enrichment analysis showed that the IPF-related processes hub genes were enriched in antigen processing and presentation of exogenous peptide antigen via MHC class II, chromosome, centromeric region, and cytoplasmic dynein complex.

In addition, module analysis conducted by MCODE plugin in Cytoscape identified several modules in the PPI network. Then, the top four significant modules were selected for subsequent analysis. A significant module, which gained the highest MCODE score, contained 24 nodes and 150 edges. Subsequent functional enrichment analysis indicated that the genes in this module were primarily enriched in antigen processing and presentation of exogenous peptide antigen via MHC class II, chromosome, centromeric region, cytoplasmic dynein complex, and type I interferon signaling pathway (Figure 4B). Fourteen genes (*DYNLL1*, *DYNC1LI1*, *DYNLL2*, *HLA-DRB5*, *HLA-DPB1*, *HLA-DQB2*, *HLA-DQA2*, *HLA-DQB1*, *HLA-DRB1*, *POLR2L*, *CENPP*, *CENPK*, *NUP133*, and *NUP107*) were simultaneously detected by both hub gene and module analysis.

## Discussion

Although the biological basis of IPF has been investigated in the past years, the cellular and molecular mechanisms of IPF are very complicated and remain unclear. In the present study, we performed the first large-scale integrative analysis of TWAS and mRNA expression profiles for IPF, which successfully detected some plausible genes as well as pathways, and can potentially provide novel insights to better understand the molecular mechanisms underlying the development of IPF.

Transcriptome-wide association study detected several significant IPF-related genes previously reported in GWAS, such as *DSP* and *MUC5B*. *DSP* encodes desmoplakin, which is an important component of the desmosome structure. Desmoplakin is involved in the mechanical linkage of cells, stabilization of tissue structure and the process of cell migration, proliferation, and differentiation. Accordingly, *DSP* is essential to cell-cell adhesion and epithelial barrier function (Vasioukhin et al., 2001). Previous evidence has suggested that *DSP* expression is higher in IPF lung than in the lungs of healthy control subjects and the intron 5 variant rs2076295 was found to be associated with decreased *DSP* expression, indicating that differential *DSP* expression plays an important role in IPF etiology (Mathai et al., 2016). *MUC5B* encodes for mucin 5B, which is produced by airway epithelial cells and is a major gel-forming mucin in the mucus. Mucin 5B is involved in the production of airway mucous and may have a significant role in mucociliary clearance and airway defense (Roy et al., 2014; Evans et al., 2016). Increased *MUC5B* expression might impair mucosal defense of host and thus lead to the reduction of lung clearance of inhaled particles, dissolved chemicals, and microorganisms (Seibold et al., 2011). The *MUC5B* promotor variant rs35705950 is a common variant that accounts for a large proportion of risk for the development of familial interstitial pneumonia and IPF (Seibold et al., 2011; Todd et al., 2015; Evans et al., 2016; Zhang et al., 2019). Interestingly, a retrospective study has demonstrated improved survival of patients with this promoter variant compared with those without this variant, indicating that this variant might be a potential prognostic indicator (Peljto et al., 2013). These paradoxical findings imply that further investigation is required to clarify the biological mechanism by which this promoter variant promotes the development of IPF.

Besides, attention should be paid to genes simultaneously detected by hub gene and module analysis, such as *DYNC1LI1*, *DYNLL1*, and *DYNLL2*, which are likely to be related with IPF but not reported earlier. *DYNLL1* and *DYNLL2* are related to cell cycle spindle assembly and chromosome separation and may be involved in the change or maintenance of the spatial distribution of cytoskeletal structures (Dunsch et al., 2012). *DYNC1LI1* is related to microtubule motor activity and may play a role in binding dynein to membranous organelles. These three genes belong to the cytoplasmic dynein subunit gene. Cytoplasmic dynein acts as a motor for the intracellular retrograde motility of vesicles and organelles along microtubules (Dunsch et al., 2012). Besides, human airway epithelium is characterized by the presence of ciliated cells bearing motile cilia, and specialized cell surface projections containing axonemes consisted of microtubules and dynein arms, providing ATP-driven motility (Tilley et al., 2015). In the airways, cilia function together with airway mucus, plays an important role in mediating mucociliary clearance and eliminating the inhaled particles and pathogens (Evans et al., 2016). Cilia dysfunction and clearance impairment would result in chronic airway inflammation and infection, bronchiectasis, and distal lung remodeling. Besides, the hub gene PPI network showed that the genes involved are mainly enriched in cytoplasmic dynein complex and antigen processing and presentation, suggesting the critical role of cilia function and immune response in the development of IPF.

Gene ontology and KEGG pathway enrichment analysis detected several candidate biological pathways for IPF, mainly involved in immune inflammation response and infection. For instance, antigen processing and presentation of peptide or polysaccharide antigen via MHC class II. Human leukocyte antigen (HLA), encoded by the human MHC gene complex, plays a critical role in the antigen presentation of peptides and the regulation of immune response (Bodis et al., 2018). A GWAS analysis has identified two risk alleles in the HLA region (DRB1^∗^15:01 and DQB1^∗^06:02) that were related to IPF (Fingerlin et al., 2016). Besides, several studies have suggested the role of HLA region in the development of IPF (Falfan-Valencia et al., 2005; Aquino-Galvez et al., 2009; Xue et al., 2011; Zhang et al., 2012, 2015). The association between HLA and IPF may suggest the potential etiologic role of autoimmunity in IPF. Recently, a nationwide retrospective cohort study in Korea involving 38,921 IBD patients and 116,763 patients without IBD suggested that patients with IBD, especially Crohn’s disease, have an increasing risk for the development of IPF (Kim et al., 2020). In addition, a 1:1 retrospective case-control study (196 IPF cases and 196 controls) has indicated that hypothyroidism, an immune-mediated process, was common among IPF patients and was found to be associated with decreased survival time as an independent predictor of mortality in IPF patients (Oldham et al., 2015). Besides, diabetes has been reported to be a risk factor of IPF (Enomoto et al., 2003; Gribbin et al., 2009). Type 1 diabetes, also known as insulin-dependent diabetes, is an organ-specific autoimmune disease. Gene variants in the HLA region have been found to be related to the susceptibility of type 1 diabetes mellitus (Nejentsev et al., 2007). Naturally, these autoimmune diseases may share genetic basis contributed by genetic variations in HLA region. Since, observational associations are prone to reverse causality and confounding, further investigation is warranted to characterize the pathophysiologic link between this genetic variation and disease.

Another primary candidate biological pathway was shown to be infections (both viral and bacterial), which is also closely related to the antigen stimulation and immune response, such as herpes simplex and Staphylococcus aureus infection. Previous studies have demonstrated that virus may be involved in disease initiation. And the presence of herpes viral DNA and epithelial cell stress in the lungs of asymptomatic relatives are at risk for the development of familial IPF (Moore and Moore, 2015). A recent meta-analysis of 20 case-control studies with 1,287 participants (634 IPF cases and 653 controls) has reported that the existence of persistent or chronic viral infections significantly associate with the increasing risk of the development of IPF, but not with the aggravation of IPF (Sheng et al., 2020). Previous studies in mice models have reported that viral infection could promote the formation of lung fibrosis (Mora et al., 2006, 2007; Qiao et al., 2009). Especially, animal experiments have been applied to provide evidence of the pathogenesis of lung fibrosis regulated by gamma herpesvirus (Moore and Moore, 2015). These animal experiments have also indicated that previous infections seem to make lung epithelial cells reprogrammed during the incubation period, producing profibrotic factors, leading to the enhanced susceptibility to subsequent fibrosis damage in lung. Nevertheless, infections in susceptible hosts or the exacerbation of existing fibrosis involve active viral replication and are affected by antiviral therapy (Moore and Moore, 2015). In addition, activated leukocyte signals in IPF patients provide further support for infectious processes driving the progression of IPF. Studies have also reported that bacterial infections play a role in the progression and prognosis of IPF. A microbiome analysis of IPF bronchoscopic alveolar lavage (BAL) samples suggested that the increase in relative abundance of two operational taxonomic units (*Streptococcus* OTU1345 and *Staphylococcus* OTU1348) was positively correlated with the progression of IPF (Han et al., 2014). In another study, reduced diversity of the lung microbiome has been found to be associated with low forced vital capacity and early mortality in patients with IPF, and a mouse model demonstrated that bleomycin-induced lung fibrosis led to a decrease in the diversity and modification of microbiota (Takahashi et al., 2018). In addition, compared with the control group, bacterial load in BAL of IPF patients has been shown to be greater. The rate of decline in lung function and the mortality risk can be partly predicted by the baseline bacterial load (Molyneaux et al., 2014). *Haemophilus*, *Streptococcus*, *Neisseria*, and *Veillonella* have found to be more abundant in cases in comparison with controls. However, animal modeling implicated that infection of *Pseudomonas aeruginosa* did not aggravate bleomycin-induced fibrosis (Ashley et al., 2014), suggesting that there might be some microbial specificity in the progression of lung fibrosis or the bleomycin-induced mouse model cannot accurately reflect the alterations of the IPF disease course induced by bacterial infection in humans. To summarize, both viral and bacterial infections may play a crucial role in the progression of IPF and may be potential predictors of disease prognosis. Additional work will be warranted to investigate the biological mechanism of infections in the progression of IPF and further explore the potential benefit of antiviral and antimicrobial therapy.

There are several limitations in our study. First, the number of genes that can be accurately imputed in the TWAS analysis is limited by the training cohort sample size, the majority of subjects were of European ancestry, and the results cannot be directly generalized to other ethnic population. Second, there may be tissue bias using lung, peripheral blood and whole blood expression reference panels. Cell-type heterogeneity within or between tissues and cross-tissue pleiotropy may introduce tissue bias. Further investigation can be implemented to address this issue if reference panels for individual cell types or states are available (Wainberg et al., 2019). Third, TWAS significant genes cannot guarantee causality, since co-regulation may lead non-causal hits. Some fine-mapping methods such as FOCUS (fine-mapping of causal gene sets) may partly address this issue, due to its ability to directly model predicted expression correlations and use them to assign genes posterior probabilities of causality (Mancuso et al., 2019). FOCUS, as a post-TWAS analysis method, can be applied on top of the genes identified by TWAS to further reduce false discoveries.

## Conclusion

In conclusion, we conducted a large-scale integrative analysis of TWAS and mRNA expression profiles for IPF. Our results provide novel insights into a better understanding of the genetic mechanism of IPF. Further functional biology studies are warranted to validate our findings and clarify the potential roles of identified genes and pathways in the development of IPF.

## Data Availability Statement

The data analyzed in this study is subject to the following licenses/restrictions: Full summary statistics for the genome-wide meta-analysis of IPF can be accessed from https://github.com/genomicsITER/PFgenetics. We would like to thank the Collaborative Group of genetic studies of IPF for providing us with the IPF GWAS summary data. The gene expression profile dataset of IPF analyzed for this study can be found in the GEO with accession number GSE110147 (https://www.ncbi.nlm.nih.gov/geo/).

## Author Contributions

ZY conceived and designed the study. WG and PG performed the statistical analysis. PG wrote the manuscript. LL, QG, and ZY provided feasible advice on data analysis and drafting manuscript. All authors contributed to the article and approved the submitted version.

## Conflict of Interest

The authors declare that the research was conducted in the absence of any commercial or financial relationships that could be construed as a potential conflict of interest.

## Footnotes

1 http://gusevlab.org/projects/fusion/

2 http://metascape.org
