# Supplementary material for: Integrative Analysis of Transcriptome-Wide Association Study and mRNA Expression Profiles Identifies Candidate Genes Associated With Idiopathic Pulmonary Fibrosis
Source: Front Genet. 2020 Dec 10;11:604324. doi: 10.3389/fgene.2020.604324 (PMC7758323; doi:10.3389/fgene.2020.604324)
Supplement: Supplementary file 2 [file Presentation_1.pdf]

Supplementary Material

1 Supplementary Figures

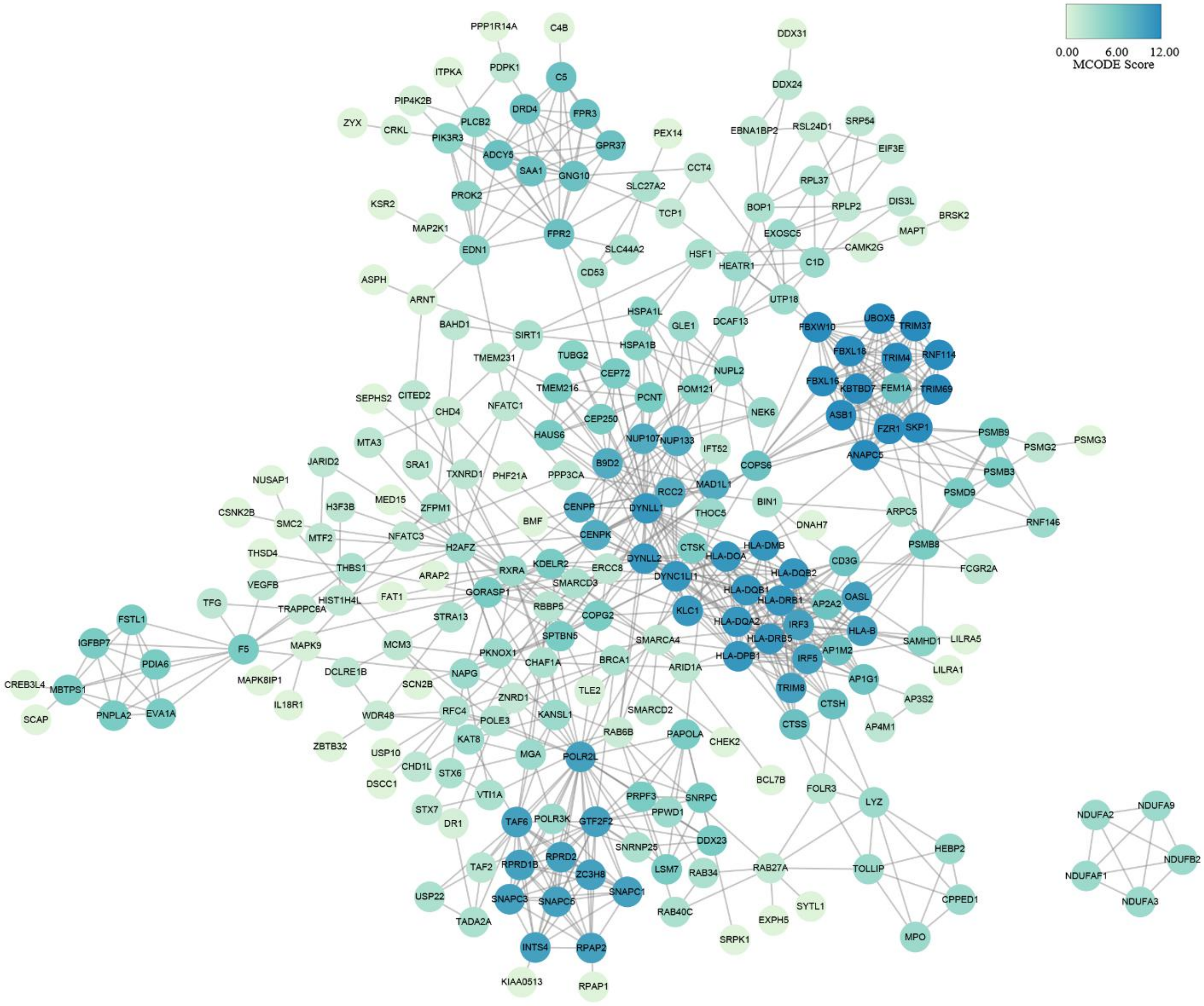

Supplementary Figure S1. Protein-protein interaction (PPI) network of significant genes identified by TWAS analysis of IPF. This PPI network contained containing 329 nodes and 893 edges. Each node represents a protein and each edge represents a protein-protein interaction.
